# *Kretzoiarctos* gen. nov., the Oldest Member of the Giant Panda Clade

**DOI:** 10.1371/journal.pone.0048985

**Published:** 2012-11-14

**Authors:** Juan Abella, David M. Alba, Josep M. Robles, Alberto Valenciano, Cheyenn Rotgers, Raül Carmona, Plinio Montoya, Jorge Morales

**Affiliations:** 1 Museo Nacional de Ciencias Naturales-Centro superior de Investigaciones Científicas (MNCN-CSIC), Madrid, Spain; 2 Institut Català de Paleontologia Miquel Crusafont, Cerdanyola del Vallès, Barcelona, Spain; 3 FOSSILIA Serveis Paleontològics i Geològics, S.L., Sant Celoni, Barcelona, Spain; 4 Departamento de Geología Sedimentaria y Cambio Climático, Instituto de Geociencias; UCM-CSIC (Universidad Complutense de Madrid-Centro Superior de Investigaciones Científicas), Madrid, Spain; 5 Departamento de Paleontología, Facultad de Ciencias Geológicas UCM (Universidad Complutense de Madrid), Madrid, Spain; 6 Departament de Geologia, Àrea de Paleontologia, Universitat de València, Burjassot, Valencia, Spain; University of California, Berkeley, United States of America

## Abstract

The phylogenetic position of the giant panda, *Ailuropoda melanoleuca* (Carnivora: Ursidae: Ailuropodinae), has been one of the most hotly debated topics by mammalian biologists and paleontologists during the last century. Based on molecular data, it is currently recognized as a true ursid, sister-taxon of the remaining extant bears, from which it would have diverged by the Early Miocene. However, from a paleobiogeographic and chronological perspective, the origin of the giant panda lineage has remained elusive due to the scarcity of the available Miocene fossil record. Until recently, the genus *Ailurarctos* from the Late Miocene of China (ca. 8–7 mya) was recognized as the oldest undoubted member of the Ailuropodinae, suggesting that the panda lineage might have originated from an *Ursavus* ancestor. The role of the purported ailuropodine *Agriarctos*, from the Miocene of Europe, in the origins of this clade has been generally dismissed due to the paucity of the available material. Here, we describe a new ailuropodine genus, *Kretzoiarctos* gen. nov., based on remains from two Middle Miocene (ca. 12–11 Ma) Spanish localities. A cladistic analysis of fossil and extant members of the Ursoidea confirms the inclusion of the new genus into the Ailuropodinae. Moreover, *Kretzoiarctos* precedes in time the previously-known, Late Miocene members of the giant panda clade from Eurasia (*Agriarctos* and *Ailurarctos*). The former can be therefore considered the oldest recorded member of the giant panda lineage, which has significant implications for understanding the origins of this clade from a paleobiogeographic viewpoint.

## Introduction

### The Fossil Record of the Giant Panda Lineage

The extant giant panda from central Asia, *Ailuropoda melanoleuca* [1], differs from other living ursids by the presence of several craniodental adaptations to durophagy (i.e., feeding tough plant material, mainly bamboo) [1], [2], [3], [4]. These adaptations are already present, to a large extent, in the Plio-Pleistocene relatives of *A. melanoleuca* (*Ailuropoda microta* and *Ailuropoda wulingshanensis*) [2], [4], which displayed a larger distribution, from northern China to Southeast Asia [5], [6], [7], than the living giant panda. Despite long-lasting disputes about the phylogenetic position of *Ailuropoda*, especially due to dietary-driven convergence in the dentition with the lesser panda (*Ailurus fulgens*) [2], [3], it is currently well-established on molecular grounds that the former corresponds to the living sister-taxon of other extant bears [8], [9], [10], [11],[12],[13], being classified in its own subfamily (Ailuropodinae) [11], [13] or tribe (Ailuropodini) [14]. Molecular data have estimated the divergence time between ailuropodines and other bears to correspond to the Early Miocene (ca. 22 mya [8], 19 mya [12] or 18 mya [2]). However, from a paleontological perspective, ailuropodine origins are still largely uncertain, due to the paucity of the available Miocene record [14]. It is generally considered that *Ailuropoda* descended from the Asian, Late Miocene ursid *Ailurarctos* [5], [7], [14]. The latter genus is first recorded by *Ai. yuanmouensis* from the Chinese locality of Yuanmou (8.2-7.2 mya) [15], being subsequently recorded by *Ai. lufengensis* from Lufeng (6.9-5.8 mya) [5], [6], [7], [14], [15]. *Ailurarctos* has been considered to be descended from an unidentified Miocene species of *Ursavus* [4], [5], [6], [7], [14], although no formal cladistic analysis had been thus far provided to substantiate such phylogenetic hypothesis.

Besides *Ailurarctos*, the extinct genus *Agriarctos*, from the Miocene of Europe [5], [16], [17], [18], has been also attributed to the Ailuropodinae. Until recently, the scarcity of available *Agriarctos* material precluded a secure assessment of its phylogenetic affinities with the Late Miocene *Ailurarctos*, which already displays incipient adaptations to durophagy, being customarily considered the oldest undoubted member of the giant panda lineage [2], [4], [5], [7], [14]. The type species of *Agriarctos*, *Ag. gaali*, was originally based on a mandibular fragment with p3-m2 [16] from the Turolian (MN12, Late Miocene) locality Hatvan (Hungary) [19]. *Ag. vighi* was also created by Kretzoi (1942), after an m1 from the Hungarian locality of Rózsaszentmárton. With no other material for comparison, provisionally we consider these two species as valid, until a more detailed study of these fossils is carried out. Although some authors attributed the holotype of *A. gaali* to *Indarctos* cf. *vireti* [19], more recently the validity of the former genus and species have been supported [20], being characterized by a strong development of the distal cusps of the premolars (usually poorly developed or absent in most of the Ursidae) and by the mesial position of the m1 metaconid [20]. *Agriarctos* was first transferred to the Ailuropodinae several decades ago [17], [18], by further including material from the Late Miocene localities of Soblay (MN10, France; [21]) and Wissbergh ( = Gau-Weinheim; MN9, Germany), previously attributed to *Ursavus depereti* [22], [23], [24]. The remains from Soblay differ from *Ursavus* by the lengthening of the upper carnassial, due to the presence of a parastyle [22], [23]. They can be therefore attributed to the genus *Agriarctos* [17], [20], even if indirectly (i.e., *Agriarctos* cf. *gaali*) (18), since a direct comparison with the *Ag. gaali* holotype is not possible due to the lack of upper dentition in the latter.

Most recently, an older, new species of this genus, *Agriarctos beatrix*, was described on the basis of dental remains from the late Aragonian (MN8, Middle Miocene) locality of Nombrevilla 2 (NOM2, Calatayud-Daroca Basin, Spain) [20], formerly attributed to *Ursavus primaevus* [25]. Given the lack of lower dentition, however, a direct comparison with *Ag. gaali* from the type locality was not feasible. On the basis of a new mandible of the same species, recovered in a similarly-aged (MN8) locality from the Abocador de Can Mata (ACM) local stratigraphic series (Vallès-Penedès Basin, Spain), here we show that *“Agriarctos” beatrix* is distinct enough as to be attributed to a different genus, *Kretzoiarctos* gen. nov. This new genus represents the oldest and most basal member of the ursid clade currently represented by *Ailuropoda*, thus being of utmost significance for understanding the origin of the giant panda lineage from both a chronological and paleobiogeographic perspectives.

### Age and Geological Background

The local stratigraphic series of ACM (els Hostalets de Pierola, Catalonia, Spain), situated in the Vallès-Penedès Basin (NE Iberian Peninsula), is a 250 m-thick stratigraphic composite succession including more than 200 fossil vertebrate localities and spanning about 1 myrs (from ca. 12.5 to 11.5 mya; late Aragonian, Middle Miocene) [26], [27], [28], [29]. The new material described in this paper (IPS46473) corresponds to an isolated find from sector ACM/C6-Camí, in a stratigraphic horizon situated 2 m above the formally-defined locality ACM/C6-A1. Based on litho- and magnetostratigraphic correlation [27], [30], both ACM/C6-A1 and the layer where IPS46473 was found are correlated to subchron C5r.2n, with an interpolated estimated age of 11.6 mya. Although no associated small mammal remains are available for IPS46473, its age indicates that this find belongs to the *Megacricetodon ibericus* + *Democricetodon crusafonti* concurrent range local biozone [29], [31], which can be correlated to the MN8 sensu Mein and Ginsburg [32]. The locality of Nombrevilla 2 (NV2), situated in the Calatayud-Daroca Basin, can be correlated to the same biozone, spanning from ca. 11.8-11.2 mya [29], [31], given the presence of *M. ibericus* and *D. crusafonti* together with the lack of the hipparionine equid *Hippotherium* [25].

### Systematic Paleontology

Order Carnivora Bowdich, 1821; suborder Caniformia Kretzoi, 1942; infraorder Arctoidea Flower, 1869; parvorder Ursida Tedford, 1976; superfamiliy Ursoidea Fischer von Waldheim, 1814; family Ursidae Fischer von Waldheim, 1814; subfamily Ailuropodinae Grevé, 1894; tribe Ailuropodini Grevé, 1894; *Kretzoiarctos* gen. nov.

Etymology: Dedicated to the paleontologist Miklós Kretzoi and from the Greek *‘arctos’* (bear).

Type species: *Kretzoiarctos beatrix* (Abella et al., 2011) comb. nov. [20].

Diagnosis: as for the type species.

### Differential Diagnosis

*Kretzoiarctos* shares many morphological characters with the other middle and late Miocene European (*Ursavus*, *Indarctos* and *Agriarctos*) and Asian (*Ailurarctos*) ursid genera, from which it is distinguished by a unique combination of features. *Kretzoiarctos* differs from *Ursavus* in the relative development of the upper premolars and lower molars. Thus, the upper carnassial in *Ursavus* is triangular and always lacks a parastyle, whereas in *Kretzoiarctos* it shows a well-developed parastyle (although not yet separated from the paracone). In turn, the lower carnassial in *Ursavus* has a well-developed sectorial blade in the trigonid, whereas in *Kretzoiarctos* the m1 displays blunt and low cuspids. Moreover, the m2 in *Ursavus* is relatively shorter than in *Kretzoiarctos* because of the lesser-developed talonid. *Kretzoiarctos* differs from *Agriarctos* in the following features: smaller dental size; less developed lower premolars without strong accessory cusps; P4 with a less developed parastyle, a less complex and more mesially-situated protocone, much less developed basal labial cingulum, more labiolingually-compressed labial cusps (parastyle, paracone and metastyle), and P4 slightly longer than the M1 (instead of being similar in length). Finally, *Kretzoiarctos* can be easily distinguished from *Indarctos* by the smaller size of the former. *Indarctos vireti* is the smallest species of this genus and the only one that could be mistaken for *Kretzoiarctos*, although they can be distinguished because *I. vireti* has no trace of parastyle and no accessory cusps in the premolars.

### *Kretzoiarctos beatrix* (Abella et al., 2011) comb. nov

Synonyms: *Ursavus depereti* (in ref. [33], p. 78); *Ursavus primaevus* (in ref. [25], p. 31); *Agriarctos beatrix* (in ref. [20], p. 188).

**Figure 1:**
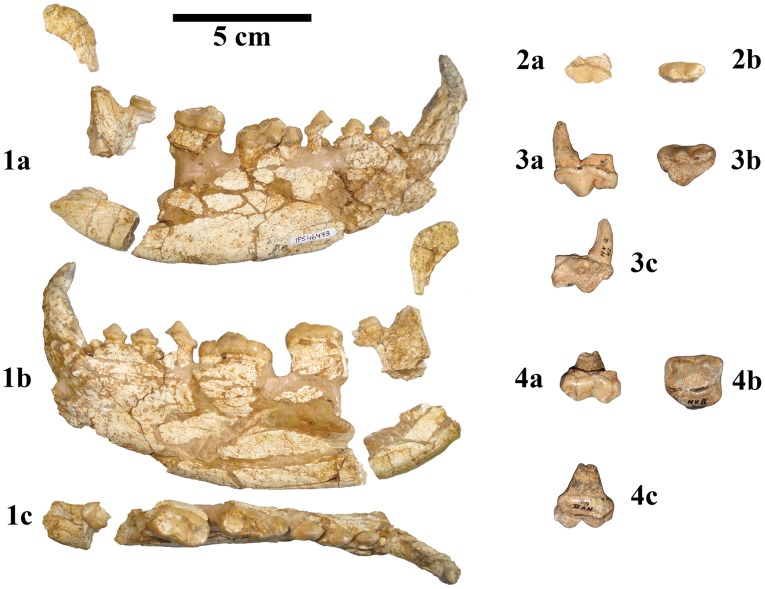
Dentognathic material of *Kretzoiarctos* gen. nov. *beatrix*. 1, Right mandible with canine and p2–m3 IPS46473 from ACM/C6-Camí in labial (a), lingual (b) and occlusal (c) views; 2, Broken P4 IPS46473 in labial (a) and occlusal (b) views; 3, Left P4 NV-2-42 (holotype) in labial (a), occlusal (b) and lingual (c) views; 4, Right M1 NV-2-42 (paratype) in labial (a), occlusal (b) and lingual (c) views from Nombrevilla 2.

Holotype: left P4, NV-2-42 (Figure 1; 3a–c) from NV II.

Paratype: right M1, NV-2-40 (Figure 1; 4a–c) from NV II.

**Figure 2:**
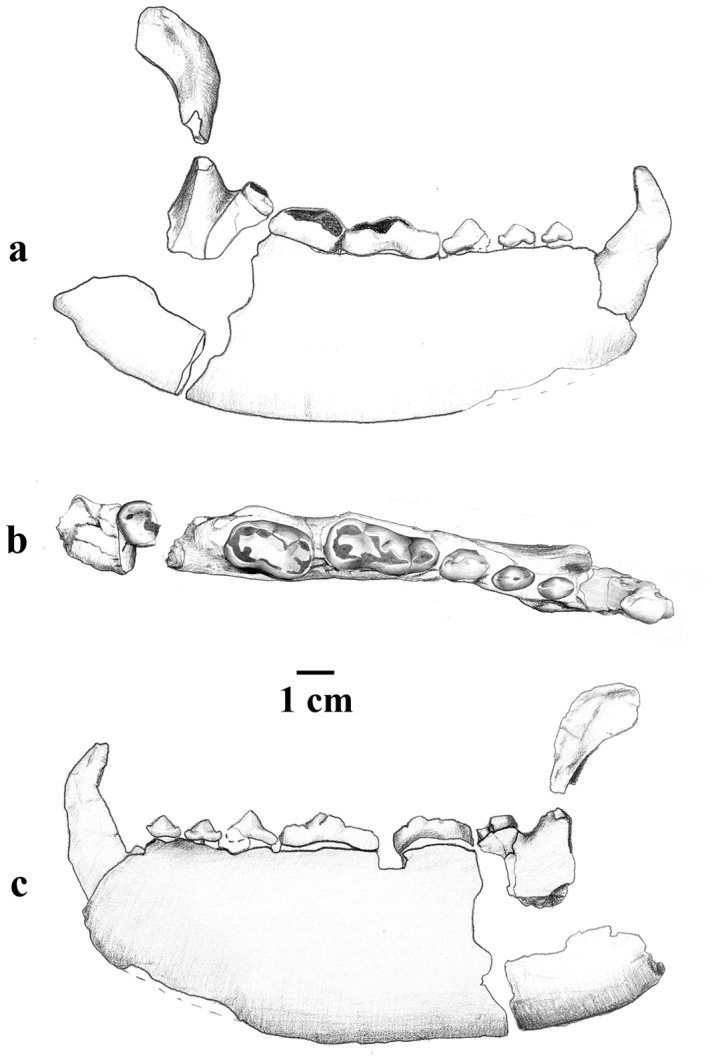
Drawing of the new material of *Kretzoiarctos* gen. nov. *beatrix* from ACM/C6-Camí. a, labial; b, occlusal; c, lingual. Artwork by Marta Palmero.

The hypodigm also includes the new material described here: partial right mandible with c1–m2 and associated P4, IPS46473 (Figure 1; 1–2 and Figure 2) from ACM/C6-Camí.

Type locality: Nombrevilla 2 (NV II; Calatayud-Daroca Basin, Spain).

Other localities: ACM/C6-Camí (Vallès-Penedès Basin, Spain).

Chronological range: the type locality has an age of ca. 11.8-11.2 mya [34], whereas ACM/C6-Camí has an estimated age of 11.6 mya, both localities thus being correlated to the MN8 *sensu* Mein and Ginsburg [32] (late Aragonian, Middle Miocene).

### Emended Diagnosis

Small-sized ailuropodine species. P4 with a well-developed protocone situated opposite to the paracone, and parastyle of moderate size but well-individualized from the protocone. M1 with a highly-developed metastyle and lingual cingulum poorly-differentiated from the protocone and hypocone [20]. Robust mandibular corpus, deepest under the m1 and m2. Low-crowned and curved lower canine. Lower premolars (p2–p4) with a single, duniform main cusp, and reduced mesial and distal accessory cusps, not separated by any diastema. Long and low-crowned m1, with the metaconid and protoconid of similar height, long and shallow talonid basin, and no cusp at the paraconid-hypoconid valley. Relativelly long m2 with well-developed trigonid and talonid basins.

### Description of the New Material

IPS46473 corresponds to a partial right mandible that preserves the canine and the lower cheek teeth (see measurements in Table 1). The mandibular corpus is short, displays a rounded ventral outline and is taller under the m1–m2 than at the symphyseal region. Despite some damage, a relatively tall and verticalized coronoid process may be reconstructed. The lower canine, which is slightly displaced out from its alveolus, is low-crowned and curved. The premolars (p2–p4) have a single main cusp that displays a duniform shape, as well as poorly-developed mesial and distal accessory cusps. There is no diastema between the lower premolars. The m1 is long and low-crowned; the metaconid and the protoconid are similar in heigh; the talonid basin is long and shallow; no cusp can be observed within the paraconid-hypoconid valley. The m2 is long, and both the trigonid and talonid basins are shallow but wide. The associated P4 is quite damaged, and only the paracone and metastyle can be observed; however, it is possible to ascertain that it was a long upper carnassial with a well-developed labial cingulum; the parastyle is preserved but it cannot be clearly observed due to breakage; both the paracone and metastyle are low and wide.

**Table 1:** Dental measurements (in mm) of *Kretzoiarctos beatrix* from ACM/C6-Camí (this study) and Nombrevilla 2 (from ref. 20).

Catalogue No.	Tooth	Length	Width
IPS46473	Right lower canine	14.73	8.84
IPS46473	Right p2	7.67	4.83
IPS46473	Right p3	5.33	8.87
IPS46473	Right p4	–	6.64
IPS46473	Right m1	22.64	10.80
IPS46473	Right m2	17.74	11.28
IPS46473	Right m3	–	9.73
NV-2-42 (holotype)	Left P4	18.5	13.0
NV-2-40 (paratype)	Rright M1	17.2	15.4

## Results

### Nomenclatural Statement

An LSID number was obtained for the new taxon (Genus *Kretzoiarctos*):

urn:lsid:zoobank.org:act:96C5EE3D-3C7B-4D5B-80FD-95C242753DFA.

### Cladistic Analysis

**Figure 3:**
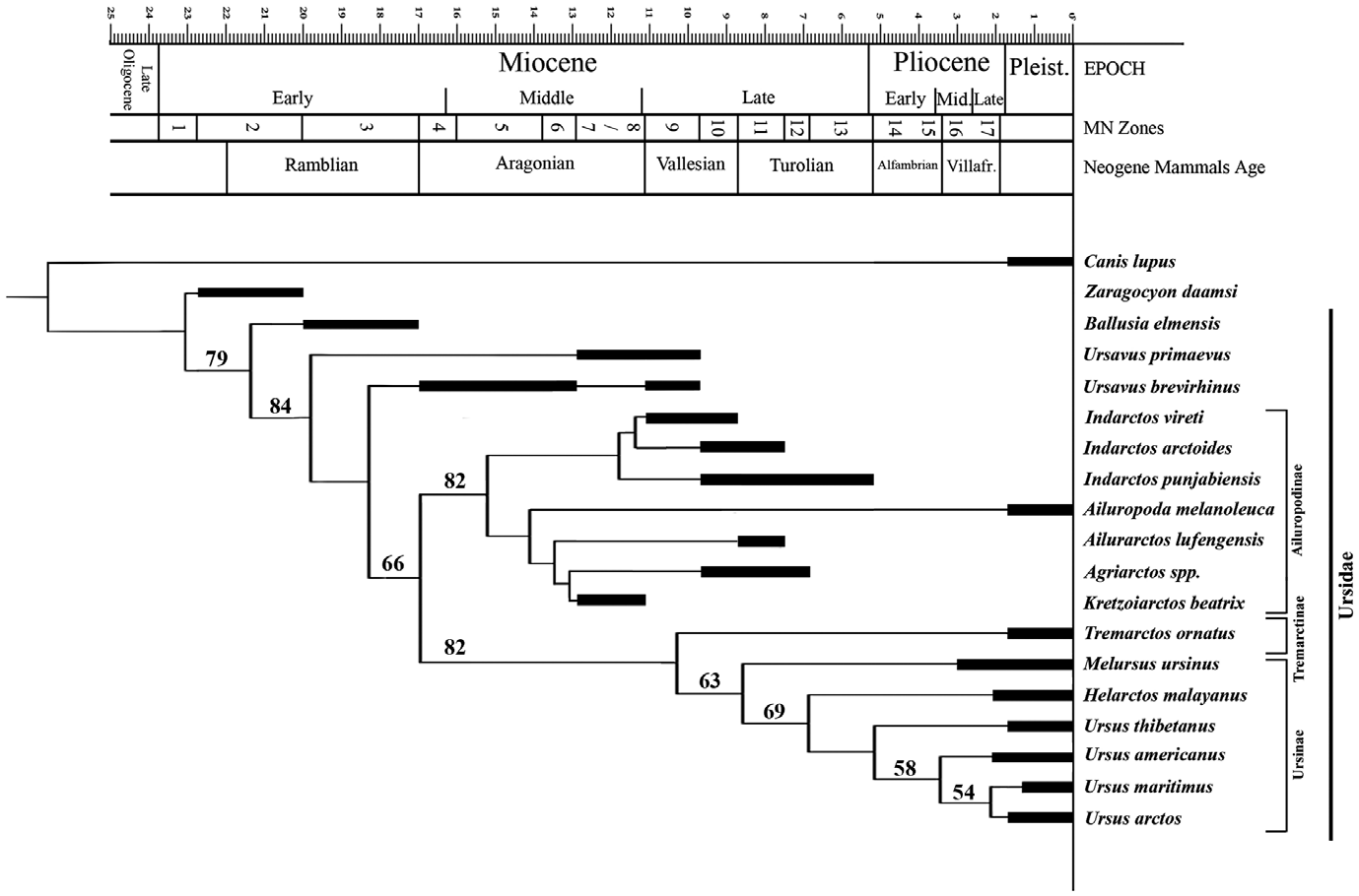
Single most parsimonious cladogram recovered by the cladistic analysis using the branch-and-bound method. See the data matrix employed in Table S1. *Agriarctos* spp. includes *A. gaali*, *A. vighi* and *A. depereti*. Numbers above each clade indicate bootstrap support over 50%. The cladogram further incorporates the known stratigraphic range for each taxon. Cladogram metrics: tree length, 159 steps; Consistency Index, 0.553; Retention Index, 0.710; Homoplasy Index, 0.447.

A cladistic analysis based on a morphologic data matrix for living and fossil ursids recovered a single most parsimonious tree of 157 steps (Figure 3). Besides the outgroup (the canid *Canis lupus*), the basal-most taxon is *Zaragocyon daamsi*, a representative of the Hemicyionidae–stem Ursoidea not included within the Ursidae [35]. The analysis therefore recovers the monophyly of the Ursidae, represented by three consecutive members of its stem lineage (*Ballusia elmensis*, *Ursavus primaevus* and *Ursavus brevirhinus*, the latter suggesting that the genus *Ursavus* is paraphyletic), and two major clades: the Ailuropodinae, including the extant giant panda (*Ailuropoda*); and the Ursinae + Tremarctinae, including all the extand ursids except *Ailuropoda*. Among the latter, the extant *Tremarctos ornatus* appears as the basal most species, followed by *Melursus ursinus.* Among the Ailuropodinae, two distinct subclades can be distinguished: that composed by *Indarctos* species (*I. vireti*, *I. arctoides* and *I. punjabiensis*), which are here included in a distinct tribe, Indarctini tribe nov. (type genus *Indarctos*); and the one including the remaining ailuropodines (tribe Ailuropodini). The Indarctini appear as the sister-taxon of the Ailuropodini, comprising the European genera *Agriarctos* and *Kretzoiarctos*, as well as the Asian *Ailuropoda* and *Ailurarctos*. The three extinct ailuropodin genera are recovered as a monophyletic clade, sister taxon of the extant *Ailuropoda*.

A bootstrap analysis (Figure 3) shows that most of the clades recovered by the most parsimonious tree are relatively well supported (i.e., bootstrap values higher than 50%). The monophyly of the Ursidae total group (bootstrap value 79) and of the Ursidae crown-group (bootstrap value 66) are well supported, whereas in contrast the successive stem position of *Ursavus* species is not supported. Among crown ursids, the dichotomy between Ailuropodinae (bootstrap value 82) and the clade composed by Tremarctinae + Ursinae (bootstrap value 82) is also well supported. Within the latter, only the position of the ursines *Helarctos malayanus* and *Ursus thibetanus* are not supported by the 50% mayority rule bootstrap analysis (see Discussion). Within the Ailuropodinae, no clade is supported by the 50% mayority rule tree recovered by the bootstrap analysis. The lack of resolution among this subfamily may be attributed to the retention of many plesiomorphies shared with both the successive stem ursids *Ursavus* and *Ballusia*, as well as with the other most primitive members of the Ailuropodinae (*Indarctos*, *Kretzoiarctos* and *Agriarctos*). However, most importantly, the inclusion of *Kretzoiarctos* and the other putative extinct ailuropodines in this subfamily is well supported not only by the most parsimonious cladogram but also by the bootstrap analysis. Accordingly, the new genus *Kretzoiarctos* emerges as the oldest member of the panda lineage (either the Ailuropodini and the Ailuropodinae) thus far recorded, thus enabling to track the fossil record of this subfamily of bears back to the MN8 (11.8-11.2 mya).

## Discussion

The results of our cladistic analysis of the Ursoidea mostly agree well with molecular analyses performed on extant ursids [13], [36], [37], especially regarding the fact that the giant panda appears as the sister-taxon of all remaining members of this family. There are a few particular points that should be taken into account. The first is the position of *Melursus ursinus*, considered a member of the Ursinae, but not showing a clear position within this clade [13]. *M. ursinus* displays a very autapomorphic craniodental morphology adapted to myrmecophagy [38], characterized by reduced teeth and even lacking several incisors. Accordingly, the basal position of *Melursus* recovered by our analysis–restricted to dental, cranial and mandibular features–even though it does not differ much from the genetic-based analysis–could be interpreted as an artifact, reflecting its very autapomorphic dietary complex relative to the remaining Ursinae. A similar anomalous result is obtained by our analysis regarding tha lack of support of the *Ursus* clade, whose monophyly is supported by molecular studies [13]. Like above, the divergent dietary adaptations displayed among the ursine bears [39] could be the cause of this ambiguity.

With regard to Ailuropodinae, our analysis indicates that this subfamily constitutes a monophyletic clade, sister-taxon to that including the remaining ursid subfamilies (Tremarctinae and Ursinae), in agreement with molecular analyses [13]. According to our results, the Ailuropodinae would be characterized by the following synapomorphies: a tall coronoid process; a high articular process; and alisphenoid canal present. Most of the remaining characteristics of this group correspond to primitive features that have been subsequently lost in both the Tremarctinae and the Ursinae, including among others: the development of the premolars; the well-developed carnassials; the wide molars; and the curved tooth row. These primitive characters are not informative for distinguishing ailuporodines from stem ursids, although they can be used to distinguish the former from the two other, more derived subfamilies of crown ursids. Along with these features, in the Ailuropodinae there is a tendency towards an increased dental complexity, as reflected by the presence of a well-developed parastyle and a complex protocone, which is a synapomorphy shared by all ailuropodines except the primitive *Indarctos vireti*. A list of all the apomorphies is given in Table S2.

The most parsimonious tree delivered by our cladistic analysis recovers two distinct ailuropodine subclades, which are here distinguished at the tribe level (Ailuropodini and Indarctini). Alternatively, these subclades could be distinguished at the subfamily rank. However, it should be taken into account that the monophyly of these tribes, unlike that of the subfamily Ailuropodinae, is not supported by the bootstrap analysis, so that merely distinguishing them at the tribe level is a more conservative option. Additional studies based on more complete material would be required in order to confirm their monophyly. If confirmed, or in case one of them was shown to occupy a more basal position than the other within the Ursidae, then it might be preferable to separate them at the subfamily level. The Indarctini includes the species of the genus *Indarctos* [40], [41], [42], which was widely distributed though Northern Africa, Eurasia and North and Central America during the Late Miocene (MN9-MN13; [40], [43]). These species include *I. vireti* (Iberian Peninsula, MN9) and *I. arctoides* (Europe MN10-11), with a relatively limited temporal range and geographic distribution, as well as *I. punjabiensis* (MN10-13), with a Holarctic distribution, and the endemic insular *I. laurillardi* from Baccinello (MN12), whose taxonomic status is yet to be determined. According to our results, the Indarctini still share several plesiomorphic traits with the Ailuropodini (non-reduced carnassials, all premolars present, wide molars, etc.), whereas some of the common characters, such as the developed pterygoideus muscles, the high coronoid process and the development of the zygomaticomandibularis muscle against the reduction of the masseteris, are derived characters present in the ursids with less meat in their diet [44]. The Ailuropodini, comprising the extant giant panda and the remaining fossil Ailuropodinae–including *Kretzoiarctos*–, can be distinguished from the Indarctini by a set of derived dental features (elongated P4, presence of a labial parastyle in the P4, development distal and mesial accessory cusps in the premolars), which we interpret as adaptations towards a more herbivorous diet. The single living representative of this group, the giant panda, markedly differs from the remaining living bears by the particular features of its masticatory apparatus [1], [2], [3], [4], which had been previously tracked backwards in time until the Late Miocene genera *Agriarctos* from Europe [16] and *Ailurarctos* from Asia [5]. This agrees well with our cladistic results, according to which the two latter genera, together with *Kretzoiarctos*, are basal members of the Ailuropodini. The new genus described here, however, further enables to push back in time the origin of the giant panda lineage to the late Middle Miocene.

### Conclusions

A new genus of extinct ursid belonging to the giant panda lineage, *Kretzoiarctos* gen. nov. (Ursidae: Ailuropodinae: Ailuropodini), is here described on the basis of new fossil remains from the Spanish site ACM/C6-Camí (Vallès-Penedès Basin). This new material allows a more precise taxonomic approach of the type material from the also Spanish locality of Nombrevilla 2 (Calatayud-Daroca Basin), previously attributed to the Late Miocene genus *Agriarctos*. With a late Middle Miocene age, *Kretzoiarctos* represents the oldest known member not only of the tribe Ailuropodini, but also of the whole subfamily Ailuropodinae (Ailuropodini + Indarctini), substantially preceding in time the other taxa that had been previously attributed to this group. Given that *Kretzoiarctos* is only known from the Iberian Peninsula (Calatayud-Daroca and Vallès-Penedès basins), a Western European origin of the giant panda lineage (Ailuropodinae) is now tentatively supported by the results of this paper. It should be taken into account, however, that the fossil record of this group is still too scarce and fragmentary, as evidenced by the various ghost lineages that must be inferred based on the Early Miocene divergence times for ailuropodines suggested by molecular data. The fossil remains of *Kretzoiarctos* reported here, however, at least conclusively document the occurrence of ailuropodines by the Middle Miocene of Eurasia, with *Ballusia* and *Ursavus* displaying a successive basal position with regard to crown ursids as a whole.

## Materials and Methods

### Cladistic Analysis

The data matrix of craniodental features employed in the cladistic analysis, including 82 characters and 19 taxa (see Table S1), was coded on the basis of original specimens of osteological and fossil material, casts, and published figures and descriptions. For *Canis lupus*, *Ursus arctos*, *Ursus americanus*, *Tremarctos ornatus*, *Ailuropoda melanoleuca, Ursavus brevirhinus*, *Indarctos vireti*, *I. arctoides* and *I. punjabiensis*, we had direct access to skulls and mandibles. For *Ursus thibetanus* and *Helarctos malayanus*, we relied on casts of mandibles and skulls. For the remaining species, we used either photographs or images from scientific papers. The character description can be consulted in the Text S1. The matrix was generated using MacClade 4.08a OS X, and was analyzed using PAUP* (Version 4.0b10 for Macintosh [45]. A maximum-parsimony analysis was performed using the branch-and-bound method, with *Canis lupus* as the outgroup. Even though *C. lupus* is not a member of the Ursidae, its cranial, mandibular and dental morphologies are supposed to be similar to the ancestor of the Arctoidea, and therefore a quite accurate choice as an outgroup for this analysis. In order to test clade robusticity, a bootstrap analysis with 1,000 replicates was performed using the branch-and-bound search option.

### Studied Material

IPS46473 is a nearly complete right mandible with canine and p2-m3 series (Figure 1; 1a-c and Figure 2) associated to a broken P4 (Figure 1; 2a–b) from ACM/C6-Camí. These specimens are housed at the Institut Català de Paleontologia Miquel Crusafont (ICP; Sabadell, Catalonia, Spain). The material from Nombrevilla 2 (NV 2) includes an upper carnassial and an M1 (Figure 1, 3–4) and it is housed in the Museo Nacional de Ciencias Naturales – Consejo Superior de Investigaciones Científicas (MNCN-CSIC), Madrid [20].

### Nomenclatural Statement

The electronic edition of this article conforms to the requirements of the amended International Code of Zoological Nomenclature, and hence the new names contained herein are available under that Code from the electronic edition of this article. This published work and the nomenclatural acts it contains have been registered in ZooBank, the online registration system for the ICZN. The ZooBank LSIDs (Life Science Identifiers) can be resolved and the associated information viewed through any standard web browser by appending the LSID to the prefix “http://zoobank.org/”. The LSID for this publication is: urn:lsid:zoobank.org:pub:B572EF80-998C-45D4-8364-14C0C7F299D9. The electronic edition of this work was published in a journal with an ISSN, and has been archived and is available from the following digital repositories: PubMed Central, LOCKSS.

## Supporting Information

Table S1 **Data matrix of craniodental features employed in the cladistic analysis, including 82 characters and 19 taxa.**
(NON)

Table S2 **List of the apomorphies found in the cladistic analysis.** Note: the changes of states are shown for each taxon. Note: Character number start in 0. Therefore Character 1 in the matrix would be character 0 in this list.
(PDF)

Text S1 **Character description.**
(PDF)
