# Supplementary material for: Kretzoiarctos gen. nov., the Oldest Member of the Giant Panda Clade
Source: PLoS One. 2012 Nov 14;7(11):e48985. doi: 10.1371/journal.pone.0048985 (PMC3498366; doi:10.1371/journal.pone.0048985)
Supplement: Table S2 — List of the apomorphies found in the cladistic analysis. Note: the changes of states are shown for each taxon. Note: Character number start in 0. Therefore Character 1 in the matrix would be character 0 in this list. (PDF) [file pone.0048985.s002.pdf]

C.lupus :

No autapomorphies

Zaragocyon :

Char. 1: 0 --> 1

Char. 25: 0 --> 1

Char. 33: 0 --> 1

Char. 43: 0 --> 1

Char. 59: 0 --> 1

Ballusia_elmensis :

No autapomorphies

Ursavus_brevirhinus :

No autapomorphies

U._primaevus :

No autapomorphies

I._vireti :

Char. 32: 1 --> 0

Char. 60: 1 --> 0

I._arctoides :

Char. 62: 0 --> 1

I._punjabiensis :

Char. 2: 1 --> 2

Char. 3: 1 --> 2

Kretzoiarctos :

Char. 1: 0 --> 1

Agriarctos :

No autapomorphies

Ailurarctos :

Char. 54: 0 --> 1

A._melanoleuca :

Char. 18: 1 --> 2

Char. 42: 1 --> 0

Char. 44: 1 --> 0

Char. 66: 1 --> 0

T._ornatus :

Char. 17: 0 --> 1

Char. 38: 0 --> 1

Char. 54: 0 --> 1

U._arctos :

Char. 38: 0 --> 1

Char. 59: 0 --> 1

U._americanus :

No autapomorphies

U._thibetanus :

Char. 4: 0 --> 1

Char. 11: 0 --> 1

Char. 77: 0 --> 1

Char. 80: 0 --> 1

Char. 81: 1 --> 0

H._malayanus :

Char. 15: 0 --> 1

Char. 65: 1 --> 0

Char. 68: 1 --> 0

Char. 70: 2 --> 1

U._maritimus :

Char. 24: 1 --> 0

Char. 31: 1 --> 0

Char. 35: 0 --> 1

Char. 57: 1 --> 0

Char. 70: 2 --> 1

M._ursinus :

Char. 4: 0 --> 1

Char. 16: 0 --> 1

Char. 18: 1 --> 0

Char. 44: 1 --> 0

Char. 51: 1 --> 0

Char. 71: 0 --> 1

Char. 73: 0 --> 1

Char. 77: 0 --> 1

Node 20 :

No synapomorphies

Node 21 :

Char. 63: 0 --> 1

Char. 66: 0 --> 1

Char. 67: 0 --> 1

Char. 68: 0 --> 1

Char. 69: 0 --> 1

Node 22 :

Char. 32: 0 --> 1

Node 23 :

Char. 29: 0 --> 1

Char. 36: 0 --> 1

Char. 70: 0 --> 1

Node 24 :

Char. 77: 0 --> 1

Node 25 :

Char. 38: 0 --> 1

Char. 73: 0 --> 1

Node 26 :

Char. 2: 0 --> 1

Char. 3: 0 --> 1

Char. 4: 0 --> 1

Char. 6: 0 --> 1

Char. 15: 0 --> 1

Char. 52: 0 --> 1

Char. 60: 0 --> 1

Char. 68: 1 --> 0

Node 27 :

Char. 26: 0 --> 1

Char. 28: 0 --> 1

Char. 43: 0 --> 1

Char. 59: 0 --> 1

Node 28 :

Char. 32: 1 --> 0

Node 29 :

Char. 33: 0 --> 1

Node 30 :

Char. 24: 1 --> 0

Node 31 :

Char. 10: 0 --> 1

Char. 14: 0 --> 1

Char. 34: 1 --> 0

Char. 57: 0 --> 1

Char. 65: 0 --> 1

Char. 70: 1 --> 2

Char. 74: 0 --> 1

Char. 78: 0 --> 1

Char. 79: 0 --> 1

Node 32 :

Char. 16: 0 --> 1

Char. 23: 0 --> 1

Char. 62: 0 --> 1

Node 33 :

Char. 18: 1 --> 0

Char. 21: 0 --> 1

Char. 73: 0 --> 1

Node 34 :

Char. 35: 1 --> 0

Char. 67: 1 --> 0

Node 35 :

Char. 22: 0 --> 1

Char. 32: 1 --> 0

Char. 40: 0 --> 1

Char. 41: 1 --> 0

Char. 55: 0 --> 1

Char. 59: 1 --> 0

Char. 75: 1 --> 0

Node 36 :

Char. 0: 1 --> 0

Char. 27: 1 --> 0

Char. 63: 1 --> 0

Char. 81: 0 --> 1

Tree 0:

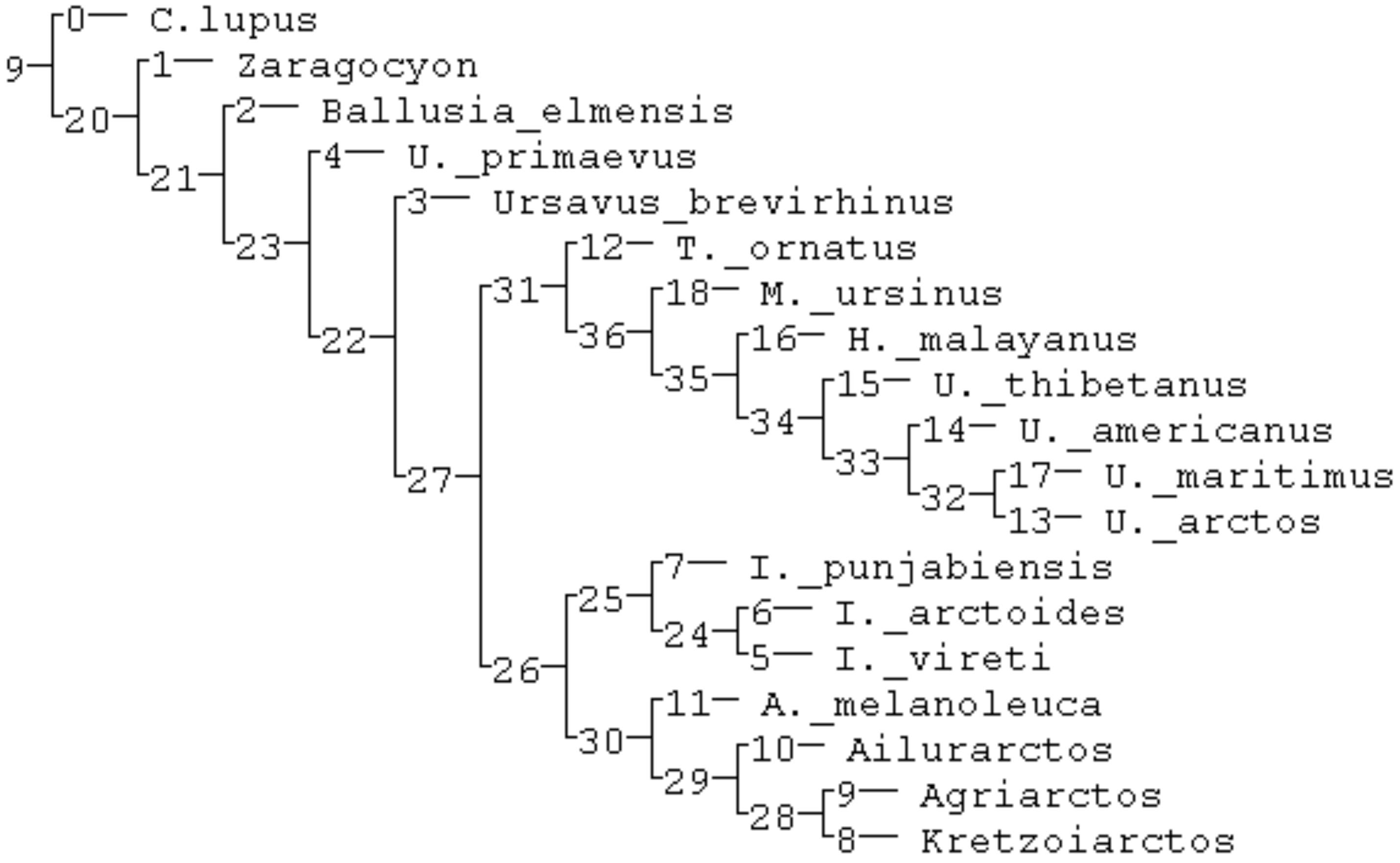
9
0 C.lupus
20
1 Zaragocyon
21
2 Ballusia_elmensis
23
4 U._primaevus
22
3 Ursavus_brevirhinus
27
31
12 T._ornatus
36
18 M._ursinus
35
16 H._malayanus
34
15 U._thibetanus
33
14 U._americanus
32
17 U._maritimus
13 U._arctos
26
25
7 I._punjabiensis
24
6 I._arctoides
5 I._vireti
30
11 A._melanoleuca
29
10 Ailurarctos
28
9 Agriarctos
8 Kretzoiarctos
