# Supplementary material for: Kretzoiarctos gen. nov., the Oldest Member of the Giant Panda Clade
Source: PLoS One. 2012 Nov 14;7(11):e48985. doi: 10.1371/journal.pone.0048985 (PMC3498366; doi:10.1371/journal.pone.0048985)
Supplement: Text S1 — Character description. (PDF) [file pone.0048985.s003.pdf]

Character Description:

1. Development in heigh of the lower canines. The canines in most of bears are not as developed as in other carnivorans. However, they display an important reduction in some of lineages such as the Ailuropodinae. This character is an important feature that may indicate herbivore adaptations.
State 0. Canine at least twice as high as the remaining maxillary and mandibular teeth.
State 1. Canine less than twice as high as the remaining maxillary and mandibular teeth.

2. Relative premolar development, compared to the remaining teeth. The premolars in most bears, especially the Ursinae, display a reduction in both number and morphology. However, there are other lineages that display the complete set of premolars or even a more complicated morphology and enamel structure.
State 0. All premolars relatively large, with developed accessory cusps.
State 1. Morphology of the premolars less complicated, without developed accessory cusps in some of them.
State 2. Both reduced morphology of the premolars and loss of at least one premolar.

3. Height of the coronoid process. The coronoid process development is a good indicator of the relationship between the *temporalis* and the *masseteris* muscles. The presence of a tall, verticalized coronoid is usually related to a more herbivorous diet.
State 0. Coronoid process does not reach twice the height of the mandibular ramus and it is relatively wide.
State 1. Coronoid process doubles the height of the mandibular ramus and it is verticalized and relatively slender.

4. Articular process position. The position of the articular process may vary among different species due to the biomechanics of the feeding complex. Therefore a higher articular process may be related to a more hypocarnivorous diet.
State 0. Same height as the tooth row.
State 1. Higher than the tooth row.

5. Articular process mediolateral width. This character that is considered to be related to the biomechanics of the lateral movement of the mandible.
State 0. Short (poorly developed).
State 1. Long (well-developed).

6. Angular process. The position of this process is also linked to the feeding complex biomechanics, and more precisely to the position and development of the *digastricus* muscle.
State 0. Short, not projected caudally and with a small insertion for the *digastricus* muscle.
State 1. Long, caudally projected and with a large insertion for the *digastricus* muscle.

7. Development of the insertion of the *masseteris superficialis* muscle in the mandible.
State 0. Large, ventrally closing the masseteric fossa.
State 1. Small, not ventrally closing the masseteric fossa.

8. Development of the insertion of the *pterygoideus* (*internus* and *externus*) muscles.
State 0. Small, not covering wide areas of the mandible.

State 1. Large, occupying large areas of the mandible, both in the articular process and in the ventrocaudal region.

9. Development of the insertion of the *digastricus* muscle.
State 0. Lined-up with the dentary bone.
State 1. Lingually projected, forming a curve towards the inner part of the mandible.

10. Development of the insertion of the *zygomaticomandibularis* muscle.
State 0. Short, not covering all the rostral part of the ascending ramus.
State 1. Long and developed. closing the whole masseteric fossa in the rostral edge.

11. Shape of the ventral outline of the jaw in lateral view.
State 0. Curved; most of the ventral outline of the dentary bone is convex in shape.
State 1. Straight.

12. Shape of the ventral outline of the jaw in lateral view, proximal to the insertion of the *digastricus* muscle.
State 0. Straight.
State 1. Forming a step between the ventral edge and the beginning of the angular process.

13. Postcanine groove in the jaw, found in the surface of the mandible, caudal to the canine.
State 0. Absent.
State 1. Present.

14. Apical morphology of the coronoid process. Even though the apex of the coronoid is somehow curved in most carnivores, its angle can be used as a character that is linked to the projection of the uppermost fibres of the *temporalis* muscle.
State 0. Slightly curved.
State 1. Apex angle close to 45º .

15. Lateral morphology of the tooth row. This character is somehow linked to the biomechanics of the feeding complex, as it affects bite strength throughout the mouth.
State 0. Curved, forming a sigmoidal shape.
State 1. Straight.

16. Jaw bone height at the level of the m1. Although this character can vary throughout the ontogeny of the animals, it can also give very important information about differences in the feeding complex and bite strength between several taxa.
State 0. Low, not reaching thrice the height of the m1.
State 1. High, exceeding more than thrice the height of the m1.

17. Mandibular symphysis ventral morphology. This character reflects the strength of the union between the two hemimandibles.
State 0. Curved.
State 1. Marked chin.

18. Premasseteric fossa. This character is only observed in some Ursoidea, such as the Hemicyonidae and the Tremarctinae; although two main types of morphologies can be detected, we code them as the same structure. In the Hemicyionidae it is located below the m1, while in the Tremarctinae it is located in a more caudal position and it is separated from the masseteric fossa by a well-developed insertion of the *zygomaticomandibularis* muscle.
State 0. Absent.
State 1. Present.

19. Position of m3. The rearrangements of the masticatory apparatus of some bears tend to modify the morphology of the ascending ramus as well as the relative position of the last lower molar in lateral view.
State 0. In front of the coronoid process, well-separated from the ascending ramus.
State 1. Included in the coronoid process, touching the ascending ramus.
State 2. Behind the coronoid process, partly or completely covered by the coronoid process.

20. Development of the distal cuspid of the i3. This character reflects its relative size compared to the incisor's main cuspid.
State 0. Small; less than half the size of the main cuspid.
State 1. Large; at least half the size of the main cuspid.

21. p1.
State 0. Present.
State 1. Absent.

22. p1-p4 diastema. This character is only observed in those species that have lost one or more premolars.
State 0. Absent.
State 1. Present.

23. p2.
State 0. Present.
State 1. Absent.

24. p3.
State 0. Present.
State 1. Absent.

25. Distal accessory cuspid of the p4.
State 0. Present.
State 1. Absent.

26. Lingual ridge of the p4, running from the main cuspid towards the lingual wall.
State 0. Absent.
State 1. Present.

27. Morphology of the paraconid of the m1 (discernible when not completely worn).
State 0. Sharp, i.e. meaning cutting edges as found in hypercarnivores.

State 1. Bunodont, i.e. meaning blunt cusps.

28. Position of the paraconid of the m1 relative to the protoconid. Although the paraconid primitively displays a mesiolingual position relative to the protoconid, most bears have switched it labially, and some (such as the Ursinae) have further closed the trigonid valley.
State 0. Mesiolingual.
State 1. Labial.

29. Heigh of the m1 protoconid relative to the paraconid.
State 0. Protoconid clearly higher than the paraconid.
State 1. Protoconid equal or subequal in heigh compared to the paraconid.

30. Morphology of the m1 protoconid. This character would be related to the action of the carnassial as an active cutting blade.
State 0. Sharp, if a clear blade is observed in the cuspid.
State 1. Bunodont, if no clear blade is observed.

31. Size of the m1 metaconid relative to the remaining trigonid cuspids.
State 0. Reduced, i.e., not individualized from the protoconid and relatively small in size.
State 1. Large, i.e., individualized from the proconid and relatively well-developed in size.

32. m1 Pre-metaconid. This cuspid, when present, displays a mesial position relative to the metaconid and a lingual position relative to the protoconid, and somehow closes the trigonid on its lingual side.
State 0. Absent.
State 1. Present.

33. Morphology of the labial part of the m1 talonid.
State 0. Narrow, without expansion.
State 1. Widened, creating an expansion.

34. Mesial angle of the m1 paraconid. It is measured in relation to the line formed by the basal-most part of the tooth crown.
State 0. High, close to 90º.
State 1. Low, clearly lower than 90º.

35. Lingual ridge of the m1 protoconid. This cristid, which runs from the protoconid towards the lingual part of the crown, is observed in some taxa together with the mesial and distal ridges.
State 0. Absent.
State 1. Present.

36. Trigonid valley of the m1. In some taxa, a closed valley is observed within the trigonid between paraconid, protoconid and metaconid.
State 0. Present.
State 1. Absent.

37. Morphology of the distal wall of the m1 trigonid.
State 0. Vertical.
State 1. Inclined.

38. Relative length of the m1 talonid relative to the trigonid.
State 0. Short.
State 1. Long.

39. m1 pre-hypoconid. This cuspid, when present, is located on the labial wall between the protoconid and the hypoconid.
State 0. Absent.
State 1. Present.

40. Morphology of the m1 crown base. In some bears, the basal portion of the crown (next to the roots) of the lower carnassials displays a rounded, swollen shape.
State 0. Straight.
State 1. Swollen, with a rounded base.

41. Morphology of the mesial ridge of the m1 paraconid in occlusal view. This character indicates the direction of the mesial cristid of the lower carnassials, which in some taxa is curved lingually.
State 0. Straight, directed towards the mesial part of the m1.
State 1. Curved, directed towards the lingual wall of the m1.

42. Morphology of the m1 talonid valley. This character whether the valley of the talonid is completely surrounded by cuspids and ridges or not.
State 0. Open, not completely surrounded by cuspids and/or ridges.
State 1. Closed, completely surrounded by cuspids and/or ridges.

43. Morphology of the m1 talonid valley. This character reflects the depth of the distal valley of the m1.
State 0. Relatively deep and forming a V-shape valley.
State 1. Shallow, not forming a v-shape valley.

44. Position of the m1 hypoconid in occlusal view.
State 0. Isolated, not forming part of the lingual cristid.
State 1. Forming part of the lingual cristid.

45. m1 distal ridge. This cristid, when well-developed, closes the talonid valley on its distal portion.
State 0. Absent or poorly-developed, not closing the talonid valley distally.
State 1. Marked, closing the talonid valley between the entoconid complex and the hypoconid.

46. Relative size of the m2 compared to the size of the lower carnassial.
State 0. Relatively small, less than half the size of the m1,.
State 1. Relatively large, at least half the size of the m1.

47. Size of the m2 paraconid in relation to the rest of the cuspids of the trigonid.
State 0. Small or absent.
State 1. Large, sub-equal in size.

48. m2 pre-metaconid. This cuspid, when present, is located mesial to the metaconid, but cannot be homologized with the paraconid,which is reduced in most bears.
State 0. Absent.
State 1. Present.

49. Relative size of the m2 talonid compared to the trigonid.
State 0. Shorter than the trigonid.
State 1. Longer than the trigonid.

50. Most developed cusp in the m2. This character reflects the relative size between the two better-developed cuspids of the m2 (protoconid and metaconid).
State 0. Protoconid.
State 1. Metaconid.

51. Development of the m2 trigonid valley compared to the total length of the talonid valley.
State 0. Small, less than half the size of the m2 talonid valley.
State 1. Large, at least half the size the m2 talonid valley.

52. m2 pre-entoconid. This cuspid is present in taxa that have a relatively complex m2 (with multiplication of cuspids), usually mesial to the entoconid.
State 0. Absent.
State 1. Present.

53. Relative development of the m2 labial cingulum relative the development of the remaining cingulids.
State 0. Small, not surrounding the whole labial wall of the m2.
State 1. Large, raning the whole labial wall (from the mesial to the distal sides) of the m2.

54. m3 development compared to the m2.
State 0. Reduced, less than ¼ the size of the m2.
State 1. Large, at least ¼ of the size of the m2.

55.
m3 morphology in mesial view.
State 0. Rounded or ovoid.
State 1. Subtriangular, with one clear tip (usually on its distal side).

56. P2.
State 0. Present.
State 1. Absent.

57. P3.
State 0. Present.
State 1. Absent.

58. P4 development relative to the M1.
State 0. Large, subequal in size.
State 1. Reduced, clearly smaller than the M1.

59. P4 parastyle.
State 0. Absent.
State 1. Present, but small.
State 2. Present but well-developed and individualized from the paracone.

60. P4 protocone relative size in occlusal view.
State 0. Small, much smaller than the remaining cusps of the trigon.
State 1. Large, similar in size with the paracone and metastyle.

61. Morphology in terms of relative morphology of the P4 protocone.
State 0. Simple, only one cusp.
State 1. Complex, with more than one cusp and/or surrounded by a cingulum.

62. Position of the P4 protocone relative to the paracone.
State 0. Mesial, close to the level of the paracone.
State 1. Distally projected, situated close to the paracone-metastyle notch level.

63. Labial expansion of the P4 at the level of the paracone-metastyle notch. This feature, when present, gives the upper carnassial a more compact shape than the usual triangular shape.
State 0. Absent.
State 1. Present.

64. Relative size of the M1 paracone and metacone.
State 0. Paracone higher than the metacone.
State 1. Both cusps sub-equal in size.

65. M1 hypocone.
State 0. Absent.
State 1. Present.

66. M1 occlusal outline.
State 0. Square (sub-equal length and breadth).
State 1. Rectangular (longer than broad).

67. M1 distal valley. This valley is situated between the hypocone and the metacone, and hence it is only observed when the hypocone is present.
State 0. Absent.
State 1. Present.

68. Development in depth of the M1 central valley, which corresponds to the trigon valley.
State 0. Deep, forming a V-shaped valley.
State 1. Shallow, not forming a V-shaped valley.

69. Relative development of the M1 lingual cingulum compared to the size of this tooth.
State 0. Well-developed,covering all the lingual wall.
State 1. Poorly developed, not covering all the lingual wall, or absent.

70. M2 relative size compared to the M1.
State 0. Reduced, similar in size or smaller than the M1.
State 1. Developed, longer than the M1.

71. Size and development of the M2 talon.
State 0. Absent or small.
State 1. Present but moderately-development, not being longer than the trigonid.
State 2. Present and well-developed, being longer than the trigonid

72. Reduction of the incisors.
State 0. Absent (all incisors present).
State 1. Present (I1 lacking and I2 reduced).

73. Enamel structure in the molars.
State 0. Smooth.
State 1. Rough.

74. Origin of the zygomatic arch in lateral view.
State 0. At the level of the M1.
State 1. At the level of the M2.

75. Rostral maximum width of the skull.
State 0. Attained at the level of the P4.
State 1. Attained at the level of the upper canines.

76. Morphology of the sagittal crest in lateral view.
State 0. Caudally projected towards the cervical vertebra.
State 1. Obliquely-inclined and moderately-developed, but not surpassing the level of the nuchal crest.
State 2. Dorsally-projected, with its highest level not situated in the dorsal-most part of the crest.

77. Development of the mastoid process. This character is related to the capability of moving the skull up and down (ventral-dorsal movement) relative to the cervical vertebrae.
State 0. Reduced, i.e., non-protruding, flattened, not surpassing the lower level of the zygomatic arch.
State 1. Wel-developed, i.e., protruding and being laterally and ventrally projected.

78. Development of the paraoccipital process.
State 0. Reduced, not surpassing the ventral level of the mastoid process.
State 1. Large and ventrally projected, longer or subequal in size compared to the mastoid process.

79. Position of the end of the hard palate in ventral view.
State 0. At about the level of the last molar.

State 1. Caudally-situated relative to the last molar (separated from it at least by the length of the M2).

80. Alisphenoid canal.
State 0. Present.
State 1. Absent.

81. Morfology of the zygomatic arch in lateral view.
State 0. Narrow, with it central portion never being wider than its rostral or caudal parts, and with a small insertion for the *zygomaticomandibularis* muscle.
State 1. Wide, with its central portion sometimes being wider than its rostral and/or caudal parts, and with an enlarged insertion for the *zygomaticomandibularis* muscle.

82.Morfology of the zygomatic arch in lateral view.
State 0. Curved, displaying a dorsally convex and ventrally concave profile, and with an increased insertion for the *zygomaticomandibularis* muscle.
State 1. Straight, with its dorsal and ventral profiles being approximately aligned with the skull's main (rostrocaudal) long axis, and without a developed insertion for the *zygomaticomandibularis* muscle.
